# Comparative Readability Analysis of Online Patient Education Resources on Inflammatory Bowel Diseases

**DOI:** 10.1155/2017/3681989

**Published:** 2017-06-27

**Authors:** Rishabh Gulati, Mohammad Nawaz, Linh Lam, Nikolaos T. Pyrsopoulos

**Affiliations:** ^1^Department of Medicine, Rutgers New Jersey Medical School, Newark, NJ, USA; ^2^Department of Medicine, State University of New York, Downstate Medical Center, Brooklyn, NY, USA; ^3^Department of Biological Sciences, University of Calgary, Calgary, AB, Canada; ^4^Division of Gastroenterology and Hepatology, Rutgers New Jersey Medical School, Newark, NJ, USA

## Abstract

**Background:**

The National Institutes of Health recommend a readability grade level of less than 7th grade for patient directed information. In this study, we use validated readability metrics to analyze patient information from prominent websites pertaining to ulcerative colitis and Crohn's disease.

**Methods:**

The terms “Crohn's Disease,” “Ulcerative Colitis,” and “Inflammatory Bowel Disease” were queried on Google and Bing. Websites containing patient education material were saved as a text file and then modified through expungement of medical terminology that was described within the text. Modified text was then divided into subsections that were analyzed using six validated readability scales.

**Results:**

None of the websites analyzed in this study achieved an estimated reading grade level below the recommended 7th grade. The median readability grade level (after modification) was 11.5 grade levels for both Crohn's disease and ulcerative colitis. The treatment subsection required the highest level of education with a median readability grade of 12th grade (range of 6.9 to 17).

**Conclusion:**

Readability of online patient education material from the analyzed popular websites far exceeds the recommended level of being less than 7th grade. Patient education resources should be revised to achieve wider health literacy.

## 1. Introduction

Recently, there has been a greater call for increased patient centeredness, patient satisfaction, and engagement as metrics for improved patient outcomes. In today's digital age, comprehensive, reliable data can be accessed on the go, quickly, and anonymously. It can be argued that the Internet is playing a major role in raising people's awareness on health problems. A study done in 2008 described that as many as 58% of the patients had used the Internet for healthcare associated information [1] with approximately 55% changing the way they thought about their health as a result of that information. In a more recent survey conducted by the Pew Research Center, 72% of Internet users looked online for healthcare information in 2011. Importantly, more than 75% began their search using a search engine like Google, Bing, or Yahoo. However, whether these available materials are sufficient and accurate enough to aid patients in their decision-making process is still debatable. The Institute of Medicine defines health literacy as “the degree to which individuals have the capacity to obtain, process, and understand basic health information and services needed to make appropriate health decisions.” According to the National Assessment of Adult Literacy (NAAL) [2], a 2003 study on the literary skills of American adults over the age of 16, approximately 36% or 77 million Americans had basic or below-basic health literacy, 53% or 114 million had intermediate level, and only 12% or 25 million had a proficient level of health literacy. It has been shown that patient compliance to treatment is better when patient literacy is taken into account and explained in a way that a patient can comprehend [3].

Readability is the ease with which a reader can understand a written text. There are many readability tests that adjudge and estimate a text's reading grade level. One of the earliest and most frequently used readability tests is the Flesch Reading Ease devised by Rudolf Flesch in 1948. Flesch Reading Ease reports a score from 0 to 100, with 90–100 intended for 11-year-old students, 60–70 for 13–15-year-olds, and 0–30 to be best understood by university graduates. After adaptation by Peter Kincaid in 1975, called the Flesch-Kincaid test, it became the Department of Defense standard with military manuals requiring adherence to specified grade levels. The New Dale-Chall readability test is regarded to be more accurate for younger readers. It calculates the grade level of a document based on sentence length and number of unfamiliar words comparing them to a list of 3,000 common words known to most 4th-grade students. The Simple Measure of Gobbledygook (SMOG) test takes into account the sentence length and the number of complex words (defined as 3 or more syllables). In a study published by Fitzsimmons et al., it was recommended that SMOG be the preferred measure of readability when evaluating consumer-oriented healthcare material [4].

With a prevalence of about 1 to 1.5 million Americans [5] and a cause-specific mortality of 51,000 in 2013 [6], Crohn's disease (CD) and ulcerative colitis (UC), collectively grouped as inflammatory bowel disease (IBD), account for a significant burden on our society. Their chronic, unrelenting nature with a potentially malignant evolutionary spectrum calls for strict patient cooperation and adherence to follow-up guidelines. It becomes essential to evaluate the appropriate readability of online health-related content for these diseases. The National Institutes of Health (NIH) recommend that patient reading material be targeted for an audience below 7th grade [7], which is denoted by a readability score of less than 7.0. In our study, we describe a comparative analysis of online patient information pertaining to Crohn's disease and ulcerative colitis through the use of multiple validated quantitative readability metrics to determine if the material is below the recommended 7th-grade reading level.

## 2. Materials and Methods

The two most commonly used English search engines, Google and Bing, were queried using the keywords “inflammatory bowel disease,” “ulcerative colitis,” and “Crohn's disease.” From the list of resulting webpages, only the first 30 searches were scanned for potential websites. As determined by click through rates, the probability of advancing beyond the first search result declines exponentially [8]. Only websites claiming to provide patient directed information, with material of at least 100 words in length and written in English, were included. Websites that required subscription or fees or that included information that was duplicitous or those authored by nonprofessional associations like Wikipedia were excluded. Patient directed information for both diseases was assimilated from the websites of the American College of Gastroenterology (ACG) [9, 10], Crohn's and Colitis Foundation of America (CCFA) [11], Mayo Clinic (Mayo) [12], National Institute of Diabetes and Digestive and Kidney Diseases (NIDDK) [13], UpToDate, Beyond the Basics (UTD) (intended for patients) [14], WebMD [15], National Health Service (NHS) [16], Patient.info [17], and New York Times Health Guide (NYT) [18]. The websites were saved as Microsoft Word (Microsoft, WA) files. All nontextual information, hyperlinks, and tables were expunged. Remaining text was edited by removal of headings, lists with bullets, and periods that did not mark the end of a sentence, such as abbreviations (e.g., Mark E. Tanchel or a.m.) and decimals. Medical terminology that was explained, proprietary words, procedures, and medications (generic or brand) were removed from the text before analysis. For example, in the sentence “your doctor may recommend a colonoscopy, which is the use of a long slender camera to evaluate the colon, or the second half of the digestive tract,” all instances of “colonoscopy” and “colon” would be removed from the text. Finally, each webpage, with the exception of UTD's Crohn's Disease article for which no clear “diagnosis” section was available, was further divided into seven subsections of Introduction, Causes, Symptoms, Diagnosis, Treatment, Surgery, and Complications. Blind assessment and modification were done by three independent reviewers who had college-level education. Website identifiers were removed, and each reviewer was provided with a heterogeneous source material to modify, so as to ensure anonymity of source information. A total of 125 subsections from 18 webpages were modified. All files were analyzed using Readability Studio (Readability Studio, OH). Analysis was conducted using six quantitative readability grade metrics: Flesch Reading Ease (FRE) [19], Coleman-Liau (CL) [20], New Dale-Chall (NDC) [21], Flesch-Kincaid (FK) [19], Gunning Fog (GF) [22], and Simplified Measure of Gobbledygook (SMOG) [23]. These algorithms take into account a multitude of factors, including but not limited to the average number of letters per word, average number of sentences per 100 words, and number of syllables per word. These metrics are detailed in Table 1. These tests (except FRE) provide a numerical grade level. Zero to 12 was interpreted as kindergarten through high school senior grade. A grade level 13 and higher was measured as their natural hierarchical undergraduate and graduate equivalents. For example, a grade level of 14.5 was interpreted as reading material intended for a college sophomore who has completed half the year. Flesch Reading Ease provides a score from 0 to 100, with 100 being material easily understood by a 4th-grade student and 0 intended for university graduate students and above. The NIH recommend that reading material be targeted to an audience below the 7th-grade level. This is interpreted as a score below 7.0 on the scales providing a grade level and scores above 80 on the Flesch Reading Ease scale. Analysis was carried out using Stat Plus software (AnalystSoft Inc., CA), Microsoft Excel (Microsoft, Redmond, WA), and Prism 6 (GraphPad Software, Inc., CA). *p* < 0.05 was considered significant for Mann–Whitney test.

## 3. Results and Discussion

**Figure 1:**
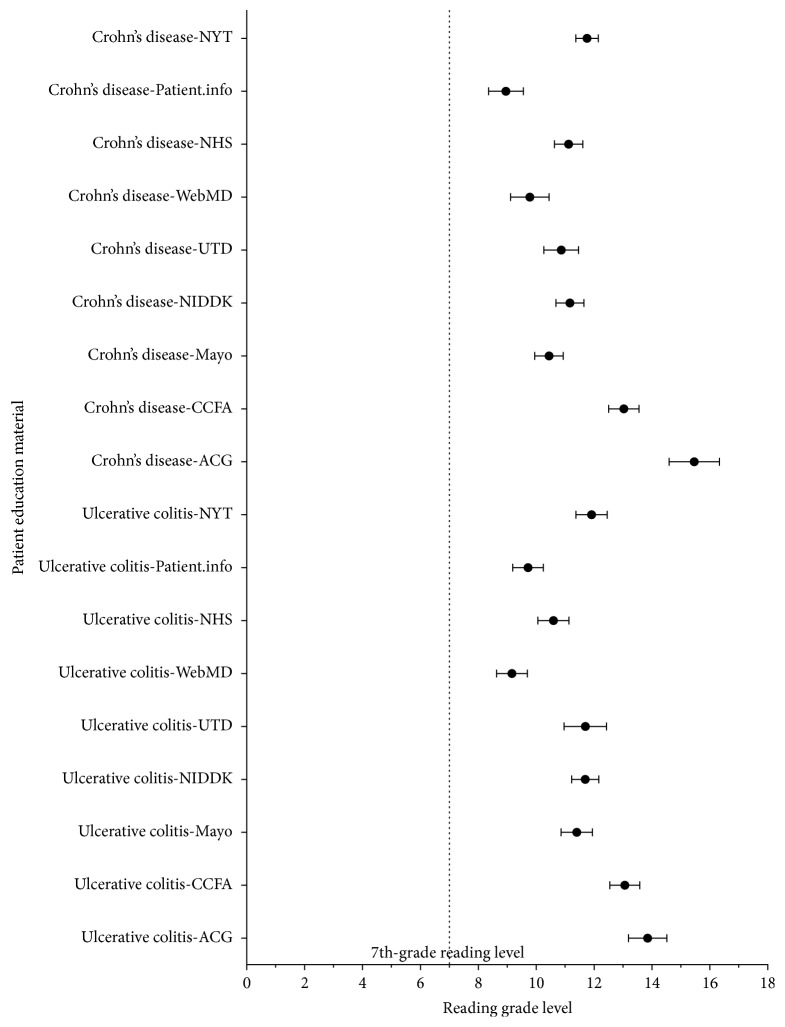
Forest plot showing the mean reading grade level with 95% confidence intervals for Crohn's disease and ulcerative colitis. The NIH recommend that the reading grade level be under the seventh-grade level. ACG: American College of Gastroenterology; CCFA: Crohn's and Colitis Foundation of America; Mayo: Mayo Clinic; NIDDK: National Institute of Diabetes and Digestive and Kidney Diseases; UTD: UpToDate, Beyond the Basics; NHS: National Health Service; NYT: New York Times Health Guide.

**Figure 2:**
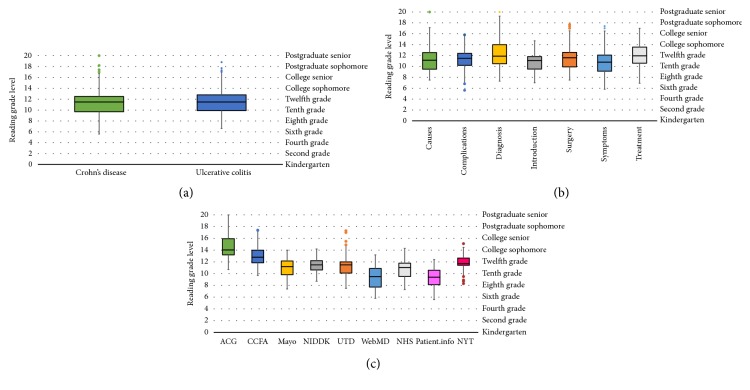
(a) Box and whisker plots depicting distribution of readability grade levels for Crohn's disease and ulcerative colitis. Median reading grade level for both diseases is 11.5. (b, c) Box and whisker plots of readability grade levels by subsection and source website, respectively. ACG: American College of Gastroenterology; CCFA: Crohn's and Colitis Foundation of America; Mayo: Mayo Clinic; NIDDK: National Institute of Diabetes and Digestive and Kidney Diseases; UTD: UpToDate, Beyond the Basics; NHS: National Health Service; NYT: New York Times Health Guide.

Analysis was carried out on modified text, comprising subsections from analyzed websites. A Forest plot analysis shows that the mean reading grade with 95% confidence interval for each website was significantly above the recommended grade level of 7.0 or below (Figure 1). The median reading grade level for Crohn's disease was 11.5 (range: 5.6 to 20), which was similar for UC with a median grade level of 11.5 (range: 6.6–18.8) (Figure 2(a)). When analyzed by subsection, it was seen that the treatment subsection required the highest degree of education with a median reading grade level of 12.0 (range: 6.9 to 17.0) (Figure 2(b)). This was closely followed by text pertaining to the diagnosis subsection with a median of 11.9 (range: 7.3–20.0). Surgical aspects of treatment had a median reading grade level of 11.6 (range: 7.5–17.7), followed by complications with a median of 11.5 (range: 5.6–15.8), causes with a median of 11.2 (range: 7.5–20.0), introduction with a median of 11.1 (range: 7.0–14.7), and finally symptoms with a median of 10.8 (range: 5.8–17.4). Assessment of individual websites for their reading grade level was then conducted (Figure 2(c)). ACG recorded the highest readability grade level, with a median of 14.1 grade levels (range: 10.7–20). This was followed by CCFA, which had a median of 12.8 grade levels (range: 9.7–17.4), followed by NYT which had a median of 11.7 grade levels (range: 8.3–15.1), NIDDK and UTD, both recording a median of 11.5 grade levels (range: 8.7–14.2 and 7.5–17.3, resp.), Mayo Clinic, which had a median of 11.2 grade levels (range: 7.4–14), NHS, which had a median of 11.1 grade levels (range: 7.3–14.3), WebMD, which had a median of 9.5 grade levels (range: 5.8–13.2), and finally Patient.info, which had a median of 9.4 grade levels (range: 5.6 to 12.4).

**Figure 3:**
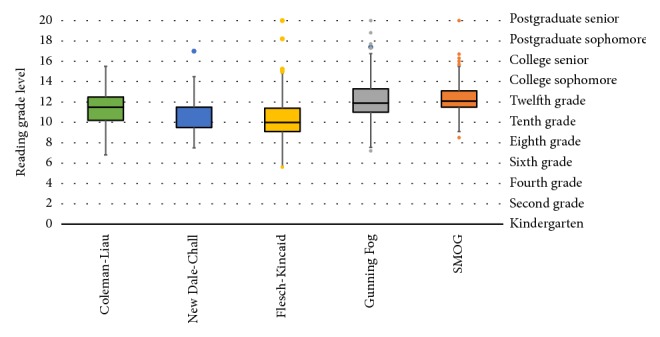
Box and whisker plot depicting distribution of readability grade levels as measured by different readability metrics. SMOG: Simple Measure of Gobbledygook.

**Figure 4:**
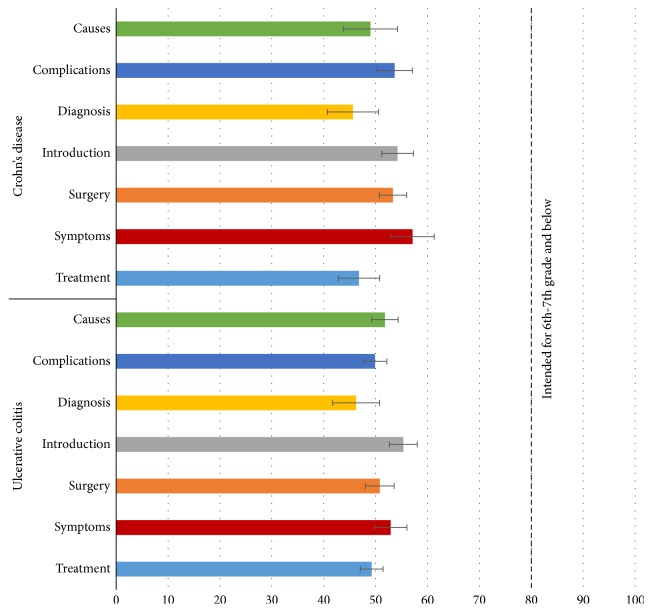
Assessment of text using FRE readability test. This figure depicts a bar graph with elevated reading grade level comprehension required for different subsections within the text. A score from 90 to 100 is intended for 11-year-old students and from 60 to 70 for 13–15-year-olds and a score from 0 to 30 is best understood by university graduates. Vertical axis at *x* = 80 approximates to 6th-grade level of comprehension. FRE: Flesch Reading Ease; ACG: American College of Gastroenterology; CCFA: Crohn's and Colitis Foundation of America; Mayo: Mayo Clinic; NIDDK: National Institute of Diabetes and Digestive and Kidney Diseases; UTD: UpToDate, Beyond the Basics; NHS: National Health Service; NYT: New York Times Health Guide.

A box and whisker plot depicting distribution of readability grade levels by the readability metric utilized is shown in Figure 3. SMOG followed by Gunning Fog registered the highest median readability grade levels with 12.1 and 11.9, respectively. These findings are supported by results of readability analysis using the Flesch Reading Ease test.  Figure 4 demonstrates the mean FRE scores by subsection, showing treatment subsection to be the most difficult with a calculated mean FRE score of 46.8 ± 4.0 and 49.2 ± 2.2 for CD and UC, respectively. On the contrary, the lowest readability grade level was demonstrated by the symptoms subsection with a mean calculated FRE score of 57.1 ± 4.2 for Crohn's disease and introduction subsection with a mean calculated FRE score of 55.3 ± 2.7 for ulcerative colitis.

**Figure 5:**
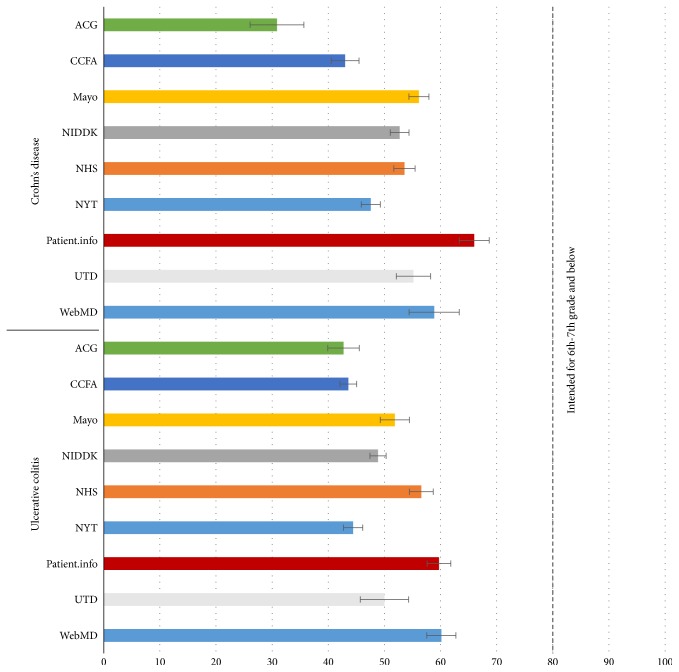
Assessment of text using FRE readability test per website, calculated by the mean FRE scores of each subsection within each website. This figure depicts a bar graph with elevated reading grade level comprehension required for different websites analyzed. A score from 90 to 100 is intended for 11-year-old students and from 60 to 70 for 13–15-year-olds and a score from 0 to 30 is best understood by university graduates. Vertical axis at *x* = 80 approximates to 6th-grade level of comprehension. FRE: Flesch Reading Ease; ACG: American College of Gastroenterology; CCFA: Crohn's and Colitis Foundation of America; Mayo: Mayo Clinic; NIDDK: National Institute of Diabetes and Digestive and Kidney Diseases; UTD: UpToDate, Beyond the Basics; NHS: National Health Service; NYT: New York Times Health Guide.

Concurring with other readability metrics, FRE reading grade estimates for Crohn's disease also provided the highest mean readability score (suggesting easier readability) for the website Patient.info (66.0 ± 2.7) and lowest estimated FRE reading score of 30.1 ± 4.8 for ACG, suggesting higher elevated reading grade (Figure 5). For ulcerative colitis, WebMD had the highest mean readability FRE score of 60.1 ± 2.6, whereas ACG scored the least mean readability FRE score of 42.7 ± 2.8.

**Figure 6:**
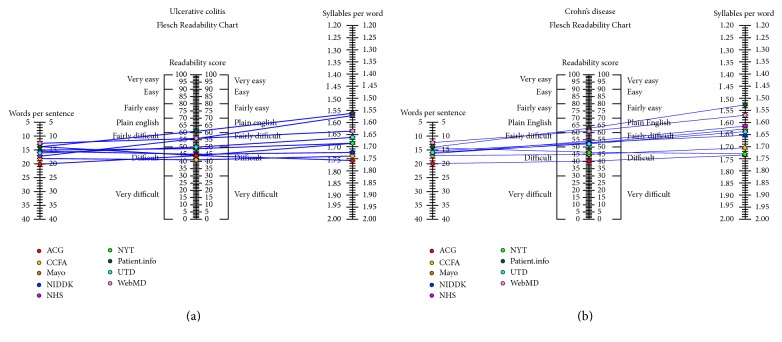
Flesch Readability Chart illustrating the components of the Flesch-Kincaid readability metric for each website of CD and UC, respectively. Each article was graded as a whole rather than the mean of the subsections under each article. Higher words per sentence and syllables per word result in a lower readability score.

This information was then examined as a measure of FRE metric components. A FRE readability analysis graph highlights the number of words and syllables per sentence for each representative website (Figures 6(a) and 6(b)). Higher mean numbers of words per sentence and syllables per word correspond to a lower readability score for FRE test. Again noticeable is the elevated difficulty for text from ACG and the relatively easier “plain English” provided by Patient.info and WebMD. This analysis was performed on text of a website as an aggregate of its component subsections.

### 3.1. Significance

Our study demonstrates that reading grade levels for both ulcerative colitis and Crohn's disease from the studied peer-reviewed websites, after modification, are substantially higher than the recommended readability grade level. Resources from the American College of Gastroenterology had the highest estimated reading grade level followed by Crohn's and Colitis Foundation of America. Treatment subsection had the highest readability grade level for both UC and CD.

Crohn's disease and ulcerative colitis are most commonly diagnosed in late adolescence and early adulthood, but they may occur at all ages [24]. It is worth noting that 52% of Internet users worldwide as of November 2014 were between the ages of 15 and 34 years, thus including a major disease cohort [25]. This transformative and stressful period is prone to external social pressure that influences and determines adult behavior. A recent study showed that only 11% of studied adolescents with IBD had adequate health-related readiness at transition from adolescent to adult-oriented healthcare systems [26]. Furthermore, as disease onset usually occurs in early adulthood [27], it can become a considerable impediment for the remainder of working life, leading to a decline in overall quality and productivity [28]. Our findings display that diagnosis and therapeutic sections from the studied websites require an elevated readability level that may be an impediment to accurate disease comprehension. Furthermore, recent therapeutic advances in IBD are increasingly targeting putative inflammatory pathways [29], with biologic treatments serving as first-line therapy in moderate to severe disease. These drugs are associated with significant adverse effects [30]. With an elevated reading grade level online, the healthcare knowledge seeking Internet consumer should be sufficiently comfortable with the complex information provided.

Crohn's disease and ulcerative colitis pose a significant burden on our society, both emotionally and economically. Anxiety and depression have been shown to have robust association with IBD [31] and are known to influence disease activity [32]. This fact can be compounded by accessing information using random links or alternative health websites [33]. According to a study done in 2012, the annual direct cost of IBD in the United States was $6.3 billion ($3.6 billion for CD, $2.7 billion for UC) [34]. It is seen that healthcare costs decline when knowledge gap about the disease declines [35]. With such staggering figures, it becomes pertinent that patients have a more thorough understanding of their conditions leading to improved healthcare outcomes.

A 2009 study investigating the quality of web-based information on IBD found that there was no relation between the position of the website on a search engine query and the quality of information provided [36]. CCFA website appeared on the first webpage for both UC and CD on our search query. Both of these websites demonstrate a readability that is suitable for a college freshman. This is partly due to greater words per sentence with higher proportion of polysyllabic words as evidenced by high SMOG and Gunning Fog levels.

### 3.2. Readability Tests

Readability is a vital yardstick for assessment of physician-patient communication. Readability tests evaluate text independent of its structure, relationship, or syntax. This allows analyses to be conducted without loss of reliability and to provide a fair estimate of used verbiage. Each test evaluates grade level based on different criteria to determine readability. A multivariate analysis of the same text using these aforementioned tests provided a realistic grade level estimate. Simpler sentence structure and word syntax should be utilized in the development of health-education materials. Substituting complex words, for example, “advantageous” to “helpful,” “regarding” to “about,” “eliminate” to “end,” and “exhibit” to “show,” can improve readability of text. The length of a sentence should be limited to 8–10 words [37]. The use of visual aid such as diagrams and illustrations should also be utilized [38]. However, the presence of complex medical terminologies, which are often long and polysyllabic, artificially inflates the suggested readability.

### 3.3. Limitations

Grade level scores tend to be less precise and should be interpreted broadly as a general range of difficulty rather than a fixed grade level. Even materials written at a low-grade level may be difficult to comprehend if proper attention is not paid to organization, layout, and design. To overcome these shortcomings, multiple readability tests were utilized which looked at varied variables. Use of multimedia and impact of graphs and images were not assessed. This study also did not take into account health information that was available in other languages.

## 4. Conclusions

The National Institutes of Health and the American Medical Association recommend that the readability level of patient education materials be written below the 7th-grade level to be effectively understood by the public. This comparative analysis of text pertaining to IBD from prominent websites demonstrates high reading grade level required for comprehension. This suggests the need to revise such information to improve health literacy and enable a better shared decision-making process.

## Figures and Tables

**Table 1:** List of formulae used by different readability grade estimation models for textual information.

Coleman-Liau Index = 0.0588L − 0.296S − 15.8
Flesch-Kincaid = (0.39 × ASL) + (11.8 × ASW) − 15.59
New Dale-Chall = 0.1579 × PDW+ 0.0496 × ASL + 3.6365
Gunning Fog = 0.4 (ASL + PHW)
SMOG = 3 +$\sqrt{PSW}$
Flesch Reading Ease = 206.835 − (1.015 × ASL) − (84.6 × ASW)

L: mean number of letters per 100 words; S: mean number of sentences per 100 words; ASL: mean number of words per sentence; ASW: mean number of syllables per word; ASL: mean number of words per sentence; PDW: percentage of words that are not on a list of words that an American 4th grader can understand; PHW: percentage of words containing 3 or more syllables; PSW: number of words containing 3 or more syllables in 10 consecutive sentences from the beginning, middle, and end; ASL: mean number of words per sentence; ASW: mean number of syllables per word.
